# Characterization of the mitogenome of *Cynoglossus Senegalensis* (Pleuronectiformes: Cynoglossidae)

**DOI:** 10.1080/23802359.2018.1524728

**Published:** 2018-10-26

**Authors:** Fantong Zealous Gietbong, Nack-Keun Kim, Sapto Andriyono, Hyun-Woo Kim

**Affiliations:** a Department of Marine Biology, Pukyong National University, Busan, Republic of Korea;; b Interdisciplinary Program of Biomedical, Mechanical and Electrical Engineering, Pukyong National University, Busan, Republic of Korea;; c Department of Marine, Fisheries and Marine Faculty, Universitas Airlangga C Campus Jl. Mulyorejo Surabaya East Java, Indonesia;; d KOICA-PKNU International Graduate Program of Fisheries Science, Graduate School of Global Fisheries, Pukyong National University, Busan, Republic of Korea

**Keywords:** Mitochondrial genome, *Cynoglossus senegalensis*, sole, Cameroon, Africa

## Abstract

The complete mitogenome of the *Senegalese tonguesole*, *Cynoglossus senegalensis* was determined by Illumina MiSeq platform. The complete mitochondrial genome of *C. senegalensis* was 16,519 bp in length. The mitochondrial genome of *C. senegalensis* showed a cynoglosidae-characteristic gene organization, in which translocation of control region to the position between ND1 and tRNA-Gln gene, and also inversions in tRNA-Gln gene from L-strand position to H-strand position. Phylogenetic analysis showed that *C. senegalensis* is most closely related to *C. sinicus* and *S.bilineatus*, which supports the previous result that genus Cynoglossidae is evolutionary paraphyletic.

The Senegalese tonguesole, *Cynoglossus senegalensis,* is commercially important species in western Africa, which is mainly distributed along the coastal waters from Mauritania to Congo (Obiekezie and Lick 1994). Unfortunately, Cynoglossus fishery including C. *senegalensis* was overexploited in many African countries and this species is currently listed as ‘Near Threatened’ by the International Union for Conservation of Nature and Natural Resources Red List of Threatened Species since 2015 (Adeofe et al. 2015). Cynoglossidae is also interesting both from evolutionary and taxonomic perspective, as the family is fast-evolving (Pardo et al. 2005) and paraphyletic (Xu et al. 2008), genetic information of *C. senegalensis* is strongly required for the management of its resources in western Africa.

We determined the complete mitogenome of *C. senegalensis* collected from the coastal water in Bamusso (4^°^00′09″ N 09^°^14′40″ E), Cameroon, Africa. Species identification and frozen storage were conducted by Fisheries and Oceanographic Research Station (IRAD Batoke), Cameroon. Molecular identification of COI region showed 99% sequence identity to *C.senegalensis* (GenBank Number: EU513631). Genomic DNA was isolated by Accuprep Genomic DNA Extraction Kit (Bioneer, Korea). Two large PCR products amplified by PCR with sequence-specific primers targeting COX1 and ITS regions were further processed into the small-sized fragments (∼350 bp) by Covaris^®^ M220 Focused-ultrasonicator (Covaris Inc., USA). Library was constructed by TruSeq^®^ RNA library preparation kit V2 (Illumina, USA) and its quality and quantity were confirmed by 2100 Bioanalyzer (Agilent Technologies, Santa Clara, CA, USA). DNA sequencing was conducted by MiSeq sequencer (2 x 300 bp pair ends). The phylogenetic tree was constructed by MEGA7 with minimum evolution (ME) method (Kumar et al. 2016).

The complete mitochondrial genome of *C. senegalensis* was 16,519 bp in length (GenBank accession; MH709122), which contains 13 protein-coding genes, 22 tRNA genes, and 2 rRNA (12S and 16S), as well as two noncoding regions; control region (D-Loop) and origin of light-strand replication (OL). Non-canonical start condons were identified in two genes (GTG in COX1 and ATT in ND3) and incomplete stop codons were found in ND2, COX2, ND2, ND3, ND4, and ND5. The mitochondrial genome of *C. senegalensis* showed a cynoglosidae-characteristic gene organization; the translocation of control region to the position between ND1 and inversion of tRNA-Gln genes from L-strand to H-strand (Mjelle et al. 2008; Kong et al. 2009; Mu et al. 2015; Shi et al. 2015; Wei et al. 2016; Shi et al., 2014a; 2014b). All 22 tRNA genes (69 bp to 77 bp) formed the typical clover secondary structures according to the prediction by the ARWEN (Laslett and Canbäck, 2008). The phylogenetic analysis of *C. senegalensis* showed *C. senegalensis* was most closely related to hat identified close to *C. sinicus* (82%) *and C. bilineatus* (82%) (Figure 1). This result corresponds to the previous result that genus Cynoglossidae is evolutionary paraphyletic (Xu et al., 2008).

**Figure 1.**
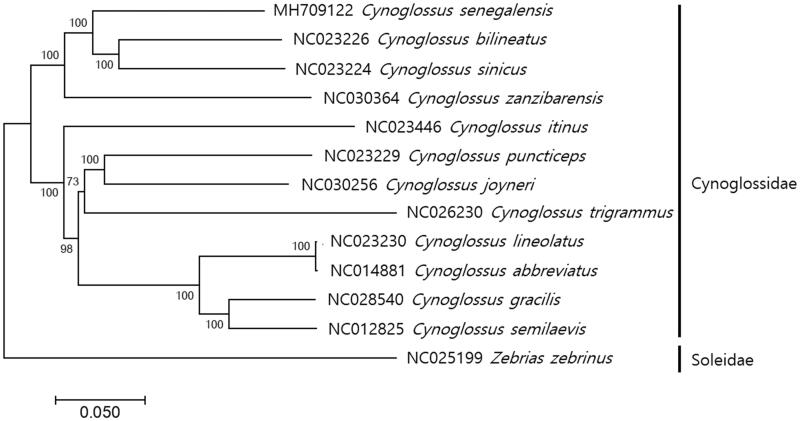
Phylogenetic tree of *Cynoglossus senegalensis*. Phylogenetic tree of complete genome was constructed by MEGA7 software with Minimum Evolution (ME) algorithm with 1000 bootstrap replications. GenBank Accession numbers were shown followed by each scientific name. The sequence data for phylogenetic analyses used in this study were as follows: *Cynoglossus senegalensi* (MH709122), *C. bilineatus* (NC023226), *C. sinicus* (NC023224), *C. zanzibarensis* (NC030364), *C. itinus* (NC023446), *C. puncticeps* (NC023229), *C. joyneri* (NC030256), *C. trigrammus* (NC026230), *C. lineolatus* (NC023230), *C. abbreviatus* (NC014881), *C. gracilis* (NC028540), *C. semilaevis* (NC012825), and *Zebrias zebrinus* (NC025199) as outgroup from Family Soleidae.
